# Five Non-motile Dinotom Dinoflagellates of the Genus *Dinothrix*

**DOI:** 10.3389/fpls.2020.591050

**Published:** 2020-11-19

**Authors:** Norico Yamada, Hiroto Sakai, Ryo Onuma, Peter G. Kroth, Takeo Horiguchi

**Affiliations:** ^1^Department of Natural History Sciences, Graduate School of Science, Hokkaido University, Sapporo, Japan; ^2^Department of Biology, University of Konstanz, Konstanz, Germany; ^3^Department of Biological Sciences, Faculty of Science, Hokkaido University, Sapporo, Japan

**Keywords:** benthic, endosymbiont, diatom, *Dinothrix paradoxa*, *Galeidinium rugatum*, *Gymnodinium quadrilobatum*, tertiary plastid

## Abstract

*Dinothrix paradoxa* and *Gymnodinium quadrilobatum* are benthic dinoflagellates possessing diatom-derived tertiary plastids, so-called dinotoms. Due to the lack of available genetic information, their phylogenetic relationship remains unknown. In this study, sequencing of 18S ribosomal DNA (rDNA) and the *rbc*L gene from temporary cultures isolated from natural samples revealed that they are close relatives of another dinotom, *Galeidinium rugatum*. The morphologies of these three dinotoms differ significantly from each other; however, they share a distinctive life cycle, in which the non-motile cells without flagella are their dominant phase. Cell division occurs in this non-motile phase, while swimming cells only appear for several hours after being released from each daughter cell. Furthermore, we succeeded in isolating and establishing two novel dinotom strains, HG180 and HG204, which show a similar life cycle and are phylogenetically closely related to the aforementioned three species. The non-motile cells of strain HG180 are characterized by the possession of a hemispheroidal cell covered with numerous nodes, while those of the strain HG204 form aggregations consisting of spherical smooth-surface cells. Based on the similarity in life cycles and phylogenetic closeness, we conclude that all five species should belong to a single genus, *Dinothrix*, the oldest genus within this clade. We transferred *Ga. rugatum* and *Gy. quadrilobatum* to *Dinothrix*, and described strains HG180 and HG204 as *Dinothrix phymatodea* sp. nov. and *Dinothrix pseudoparadoxa* sp. nov.

## Introduction

Dinoflagellates are aquatic unicellular eukaryotes, of which approximately 2,400 species have been described (Gómez, 2012). Most of them are marine planktonic photosynthetic or heterotrophic protists, however, about 160 species (50 genera) live in marine benthic environments, e.g., sandy beaches, tidal pools, on seaweed, inside and outside of corals, and within the seabed (Hoppenrath et al., 2014). Many benthic dinoflagellates have similar morphologies: their *epitheca* are small or almost unrecognizable, and cells are compressed laterally or dorsoventrally (Hoppenrath et al., 2014), facilitating motility between substrata. Some benthic dinoflagellates appear to have abandoned the active swimming lifestyle. These dinoflagellates spend most of their lives as non-motile cells without flagella, e.g., *Halostylodinium arenarium* (Horiguchi et al., 2000), *Pyramidodinium* spp. (e.g., Horiguchi and Sukigara, 2005), *Spiniferodinium* spp. (e.g., Horiguchi and Chihara, 1987), and *Stylodinium littorale* (Horiguchi and Chihara, 1983).

In this study, we focus on three non-motile benthic dinoflagellates: *Dinothrix paradoxa*, *Galeidinium rugatum*, and *Gymnodinium quadrilobatum*. The dominant phase of these species is the non-motile cell without flagella, and cell division occurs in this phase. Swimming cells are produced during cell division and released as motile daughter cells. However, the motile phase persists for only a few minutes to several hours, and then directly returns to the non-motile cell phase by development of a species-specific shaped cell wall (Pascher, 1914, 1927; Horiguchi, 1984; Horiguchi and Pienaar, 1994; Tamura et al., 2005). Molecular phylogeny and cell ultrastructures have clarified that all three species possess organelle-retaining diatom-derived tertiary plastids (ODPs; Horiguchi, 1984; Horiguchi and Pienaar, 1994; Tamura et al., 2005). Sharing this distinctive life cycle and possessing ODPs suggests their close relationship. Their exact relationship, however, is so far unknown due to the lack of genetic information on *D. paradoxa* and *Gy. quadrilobatum*.

Molecular phylogeny has confirmed that all dinoflagellates possessing ODPs belong to the family Kryptoperidiniaceae (Chesnick et al., 1997; Gottschling et al., 2017), except for several dinoflagellates, for which there is a lack of genetic information, including *D. paradoxa* and *Gy. quadrilobatum*. Due to their plastid origins, the Kryptoperidiniaceae are known as “dinotoms” (Imanian et al., 2010). The ODPs in dinotoms have two unique features that have never been reported in any other extant eukaryotes. The first characteristic is that the ODPs maintain almost all organelles of diatoms (as the name indicates). Therefore, each single dinotom cell contains two origins of nuclei, endoplasmic reticulum (ER), Golgi, ribosomes, mitochondria, and plastids, which are derived from either of diatoms or dinoflagellates (Tomas et al., 1973; Jeffery and Vesk, 1976; Horiguchi and Pienaar, 1991), although the dinoflagellate plastids lost the photosynthetic ability (Dodge, 1971; Hehenberger et al., 2014). A dinoflagellate-derived single membrane (symbiosome membrane; Bodył, 2018; Yamada et al., 2019) separates the diatom organelles from the host dinoflagellate cytoplasm. The diatom nucleus, mitochondria, and plastids of dinotoms are transcriptionally active (Imanian and Keeling, 2007; Hehenberger et al., 2014); the diatom nucleus, in particular, remains transcriptionally intact (Hehenberger et al., 2016). The second unique property of ODPs is the variety in their origins and evolutionary integration stages: ODPs are derived from at least 14 diatom species (Horiguchi and Takano, 2006; Takano et al., 2008; Čalasan et al., 2017; Yamada et al., 2017). Some ODPs have already evolved into permanently-maintained stages (Tippit and Pickett-Heaps, 1976; Figueroa et al., 2009), while other ODPs remain only temporarily as kleptoplastids, i.e., host dinoflagellates lose ODPs after a time, and need to feed repeatedly on free-living diatoms (Kempton et al., 2002; Yamada et al., 2019). Although it seems that the variation of host dinoflagellates with respect to their habitat environments, life cycles, and diatom preferences is crucial for understanding the evolutionary processes of the ODPs, many dinotoms, with the exception of *Durinskia* cf. *baltica* and *Kryptoperidinium cf. foliaceum*, have seldomly been studied following their initial descriptions.

Here, we focus on two rarely-studied, non-motile dinotoms: *D. paradoxa* and *Gy. quadrilobatum*, and confirm that they are closely related to another non-motile dinotom, *Ga. rugatum*. Furthermore, we discover two novel benthic dinotoms, which also produce dominant non-motile cells without flagella. These two species, strains HG180 and HG204, have been shown to form a clade with *Ga. rugatum* with 100% bootstrap support (Yamada et al., 2017). We identify the morphologies and ultrastructures, and define the life cycles, in order to determine the phylogenetical relationships of these five non-motile dinotoms.

## Materials and Methods

### Sample Collection and Isolation of Dinoflagellates

Sand samples containing *Gy. quadrilobatum* and HG180 were collected from sandy beaches in Japan (Table 1). Each sample was placed in a plastic cup and enriched with Daigo’s IMK culture medium (Nihon Pharmaceutical Co. Ltd., Tokyo, Japan) for culturing. The culture temperature was set to the water temperature at the sampling location (20 or 25°C), and illumination was set to 50 μmol photons m^2^/s with a 16:8 h light: dark cycle (day 8:00 to 24:00; night 0:00 to 8:00). The crude cultures of natural samples were checked daily for 2 weeks for the occurrence of dinoflagellate cells. Target cells were isolated using a capillary pipette, with several rinses in sterilized IMK medium under an inverted microscope. Each cell was then individually transferred into a 24-well plate, for establishing clonal cultures.

**Table 1:** Sampling sites and type localities of *Dinothrix* spp.

Species name	Strain	Habitat	Basionym	Type locality	Sampling site in this study
*Dinothrix paradoxa* (type species)	–	T	–	Helgoland, Germany (from an aquarium)^1^	Miura, Kanagawa, Japan (35°11'41''N, 139°35'41''E)
*Dinothrix phymatodea*	HG180	S	–	Hanashiro, Okinawa, Japan	Type locality (26°06'51.9''N, 127°44'35.8''E)
*Dinothrix pseudoparadoxa*	HG204	T	–	Marina Beach, Kwazulu-Natal, South Africa	Type locality (30°56'30.1''S, 30°18'16.6''E)
*Dinothrix quadrilobata*	–	S	*Gymnodinium quadrilobatum*	Amanzimtoti, Kwazulu-Natal, South Africa	Ishigaki, Okinawa, Japan (30°44'34.1''N 130°59'42.6''E)
*Dinothrix rugata*	HG249	S	*Galeidinium rugatum*	Mechercher Island, Palau	–

T, tidal pool; S, sandy beach.

1 The ecological type locality of *Dinothrix paradoxa* is unknown.

For tidal pool dinoflagellates, *D. paradoxa* and HG204, water samples were collected from tidal pools in Japan or South Africa, respectively, while they formed blooms (Table 1). Motile cells of *D. paradoxa* and HG204 were isolated individually using a capillary pipette. Using this method, we succeeded in obtaining stable cultures of strains HG180 and HG204; however, we failed to establish cultures of *D. paradoxa* and *Gy. quadrilobatum*. The established cultures of the HG180 and HG204 were maintained at 25°C, with illumination at 50 μmol photons m^2^/s, with a 16:8 h light: dark cycle.

### DNA Extraction and PCR Amplification

DNA extractions were performed using the QuickExtract FFPE RNA Extraction Kit (Epicenter, Wisconsin, United States) for all species except *D. paradoxa.* For *D. paradoxa*, we used the benzyl chloride method (Zhu et al., 1993).

The QuickExtract FFPE RNA Extraction Kit: 5–10 dinoflagellate cells were isolated under an inverted microscope and transferred into 10 μl of QuickExtract FFPE solution. The solution was heated at 56°C for 1 h, followed by 98°C for 2 min. One microliter of the solution was used as template DNA for the PCR. We conducted the nested PCR for 18S rDNA and the *rbc*L gene under the following conditions: an initial denaturation cycle at 94°C for 1 min, followed by 40 cycles of denaturation at 94°C for 30 s, annealing at 53°C for 30 s, and an extension at 72°C for 30 s. The final extension cycle was at 72°C for 7 min. For the first amplification, primer pairs, SR1b and SR12b, for 18S rDNA of host dinoflagellates (Supplementary Table 1; Nakayama et al., 1996) and DiatrbcL1 and DiatrbcL6 for *rbc*L gene of ODPs (Supplementary Table 1; Tamura et al., 2005) were used. The second amplification was performed using 1 μl of the first PCR amplicon, with the following primers: SR1b and SR3, SR2spin and SR7, SR4 and SR9p, SR6 and SR11, and SR8 and SR12b for 18S rDNA (Supplementary Table 1; Nakayama et al., 1996; Yamada et al., 2014), and DiatrbcL1 and DiatrbcL3; DiatrbcL2 and DiatrbcL5; and DiatrbcL4 and DiatrbcL6 for the *rbc*L gene (Supplementary Table 1; Tamura et al., 2005). The second PCR amplicons were purified and sequenced using an ABI PRISM Big Dye Terminator (Applied Biosystems, Foster City, United States). The sequence reactions were run on a DNA autosequencer ABI PRISM 3730 DNA Analyzer (Applied Biosystems, Foster City, United States).

The benzyl chloride method: since we failed to sequence the both genes of *D. paradoxa* with the QuickExtract FFPE RNA Extraction Kit, the benzyl chloride method was performed for this species. The extracted DNA of *D. paradoxa* was amplified under the same PCR condition mentioned above, with same primer pairs of the second amplification.

### Molecular Phylogenetic Analysis

All sequences were aligned by ClustalW in MEGA 7 (32-bit for mac OS; Kumar et al., 2016). As outgroups for each phylogenetic analysis, 18S rDNA of *Gymnodinium fuscum* (AF022194; type species of genus *Gymnodinium*), *Blastodinium spinulosum* (HQ226072), and *Blastodinium contortum* (DQ317536) for host dinoflagellate phylogeny, and *Bacillaria paxillifer* (HG912491) and *Eunotia naegelii* (KF733443) for the *rbc*L gene analysis for the ODPs, were used. The aligned sequences were analyzed by the maximum likelihood method using PhyML 3.0 beta version (Guindon et al., 2010) or IQTREE (Nguyen et al., 2015) with additional bootstrap analyses (1,000 replicates). The selected models for maximum likelihood PhyML analysis by the Akaike Information Criterion were the GTR + G (for 18S rDNA of host dinoflagellates) or the GTR + G + I (for the *rbc*L gene of the ODPs). The automatically-selected models for maximum likelihood IQTREE analysis were the TN + F + I + G4 (for 18S rDNA of host dinoflagellates) or the GTR + F + I + G4 (for the *rbc*L gene of the ODPs) by the Baysian Information Criterion. For bootstrap analyses of IQTREE trees, we used the ultrafast bootstrap (Minh et al., 2013).

### Light and Laser Confocal Scanning Microscopy

Four species of non-motile dinotoms were observed under light microscopy (LM; Zeiss Axioskop2 Plus light microscope; Zeiss Japan, Tokyo, Japan). Photographs were taken with a Zeiss AxioCam ERc 5 s.

The two cultured dinotoms, strains HG180 and HG204, were observed with confocal laser scanning microscopy (CLSM; Zeiss LSM 700; Zeiss, Oberkochen, Germany), to confirm the morphologies and the number of diatom nuclei. Each dinotom sample was centrifuged for 5 min at 3,000 *g* (Centrifuge 5415 D; Eppendorf, Hamburg, Germany). After removing the supernatant, 125 μg/ml of 4′,6-diamidino-2-phenylindole (DAPI; Carl Roth, Karlsruhe, Germany) was added to sample, with an ethanol and acetic acid mixed buffer (ratio 3:1, pH 6.8) for a final concentration of 6% (v/v). Staining developed over 20 min in darkness prior to CLSM observations.

### Scanning Electron Microscopy

Because of the rarity of swimming cells in strains HG180 and HG204, two different scanning electron microscopy (SEM) protocols were used; one for stable cell numbers (for non-motile cells) and one for a single-cell protocol (for swimming cells).

#### Non-motile Cells

Dinoflagellate cells were collected by centrifugation at 300 *g* (MC-100 Tomy, Tokyo, Japan). The collected cell pellet was fixed in Lugol’s solution (100 g KI, 50 g I_2_, and 100 ml glacial acetic acid in 1 L DW), which was diluted with IMK medium to the 0.4% final concentration. The fixation time was 1 h at room temperature (24°C). After rinsing, once in sterilized culture medium then twice in distilled water, for 10 min each, the sample was placed on a SEM glass plate (Ohken Shoji, Tokyo, Japan) coated with poly-L-lysine. The cells were allowed for 10 min to attach with the SEM plate. The sample on the SEM plate was then gradually dehydrated with an increasing series of ethanol concentrations (25, 30, 50, 70, 80, 90, and 95%) for 10 min each. Finally, the sample was dehydrated in 100% ethanol twice, each for 30 min, and then critically point dried (Hitachi HCP-2, Tokyo, Japan). After sputter-coating with gold for 120 s at 15 mA (Hitachi E-1045, Tokyo), the sample was observed with a SEM (S-3000 N, Hitachi, Tokyo, Japan).

#### Swimming Cells

We prepared a half-cut pipette tip (1,000 μl) with a micro pore membrane filter (3.0 μm pore size, Millipore, Cork, Ireland) attached to the one side. About 2% OsO_4_ diluted in IMK medium was added to the half-cut pipette tip, and then dinoflagellate swimming cells isolated using capillary pipettes were transferred into the pipette tip for fixation. The fixation time was for 3 min at room temperature (24°C). The sample was rinsed once in sterilized IMK medium, and twice in distilled water, for 10 min each, and then moved to the dehydration step. From the dehydration, the same protocol was followed as for non-motile cells.

### Transmission Electron Microscopy

Non-motile cells of strains HG180 and HG204 were collected by centrifugation for 5 min at 3,000 *g* (Centrifuge 5,415 D; Eppendorf, Hamburg, Germany). After removing the supernatant, the samples were placed on a gold-plated copper high-pressure freezing planchette, coated with 15 μl soy-lecithin (0.15%) in chloroform. The samples were frozen instantaneously with the planchette by a high pressure freezing device (Leica ICE; Leica, Wetzlar, Germany) at 2,100 bar. The frozen samples were post-fixed in a glass jar containing 2% OsO_4_ dissolved in water-free acetone, which was pre-cooled to −90°C. The jars were kept at −90°C for 120 h, and then gradually warmed to −30°C over 24 h; kept at −30°C for 5 h, then warmed to 0°C over 7 h. Finally, the samples were rinsed twice in 100% acetone at 0°C for 1 h each, and once more at room temperature (24°C) for 1 h. The dehydrated samples were then infiltrated with Spurr’s resin (Spurr, 1969) by gradually increasing the concentration of resin in 10% increments over 1 day. Once 100% resin was attained, the samples were left overnight. After then the samples were transferred to freshly prepared resin for 2 h, and polymerized in an oven at 65°C for 48 h. An ultramicrotome (Leica EM UC7; Leica, Wetzlar, Germany) was used for sectioning. Sections were picked up on formvar-coated three-slot grids, and stained by the method of Reynolds (1963). The samples were observed using a Zeiss EM912 Omega transmission electron microscope (TEM; Zeiss, Oberkochen, Germany).

### Life Cycle Observation

Six single cells of HG180 and HG204 were individually isolated with a capillary pipette under an inverted microscope, and transferred to a 24-well culture vessel. Isolated cells were checked daily for 2 weeks in the morning (9:00 AM) and evening (6:00 PM) to determine the time of cell division and potential transformation of daughter cells to swimming cells. The cell division rates were calculated by counting the cell numbers under an inverted microscope, once a week for 2 weeks.

## Results

### Molecular Phylogenies of Five Non-motile Benthic Dinotoms

**Figure 1:**
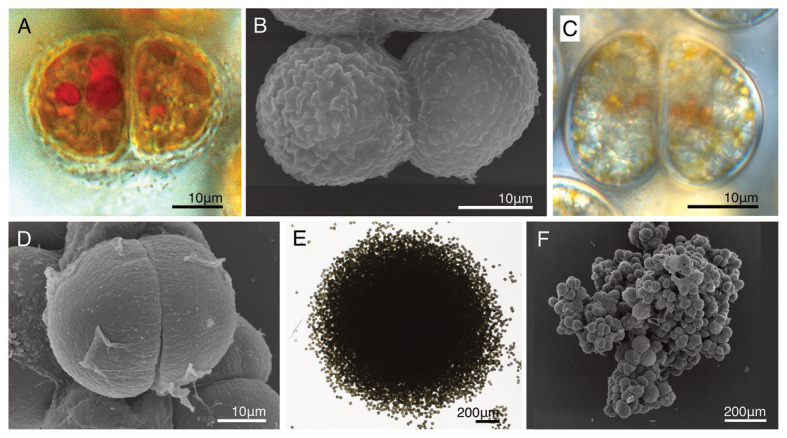
Dinoflagellate tree constructed by PhyML based on 18 rDNA. *Gymnodinium fuscum* (AF022194), *Blastodinium spinulosm* (HQ226072), and *Blastodinium contortum* (DQ317536) were used as outgroups. Numbers at the major nodes represent maximum likelihood (1,000 pseudoreplicates) bootstrap values. Only bootstrap values ≥70% are shown. GenBank accession numbers follow taxon names.

In two molecular phylogenies of host dinoflagellates, based on 18S rDNA, *D. paradoxa*, *Ga. rugatum*, and *Gy. quadrilobatum*, and the two undescribed species, HG180 and HG204, formed a clade with 100% bootstrap support within the family Kryptoperidiniaceae (Figure 1; Supplementary Figure 1). The 18S rDNA sequences differed by 0.709% (*Gy. quadrilobatum*), 0.887% (HG180), 1.182%, (*Ga. rugatum*), and 1.301% (HG204), compared to *D. paradoxa* (full length: 1,691 bp). Three strains of the dinotom *Kryptoperidinium* spp. were positioned as the sister clade of these non-motile five dinotoms with 100% bootstrap support in both phylogenies. The type species of the genus *Gymnodinium*, *Gy. fuscum* did not form a clade with *Gy. quadrilobatum*.

**Figure 2:**
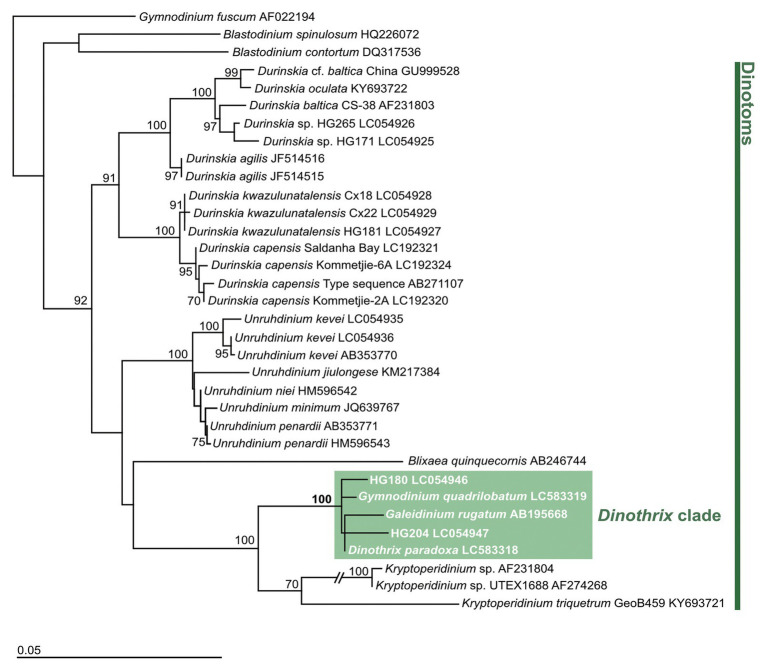
Diatom tree constructed by PhyML based on *rbc*L gene. *Bacillaria paxillifer* (HG912491) and *Eunotia naegelii* (KF733443) were used as outgroups. Bold type indicates the ODPs of dinotoms. Numbers on the major nodes represent maximum likelihood (1,000 pseudoreplicates) bootstrap values. Only bootstrap values ≥50% are shown. GenBank accession numbers follow taxon names.

A molecular phylogeny of the ODPs and free-living diatoms, inferred from the *rbc*L gene, showed that four of them formed a monophyletic clade with an undescribed *Nitzschia* species (strain KSA0120), with 86% by PhyML 3.0 beta version or 99% by IQTREE bootstrap values (Figure 2; Supplementary Figure 2). Their ODPs all belonged to the small clade of *Nitzschia sensu lato*, but the *rbc*L sequences were significantly diverse to each other: 1.179% (HG204), 1.548% (HG180), 4.348%, (*Ga. rugatum*), and 4.356% (*Nitzschia* sp., strain KSA0120, full length: 1,125 bp), compared to the ODP of *D. paradoxa* (full length: 1,357 bp, excluding a 25 bp unsequenced mid-section). We did not succeed in sequencing any ODP-encoded genes of *Gy. quadrilobatum*; therefore, its phylogenetic position remains unknown.

### The Cell Behavior of *Dinothrix paradoxa* and *Gymnodinium quadrilobatum* During Temporary Cultures

**Figure 3:**
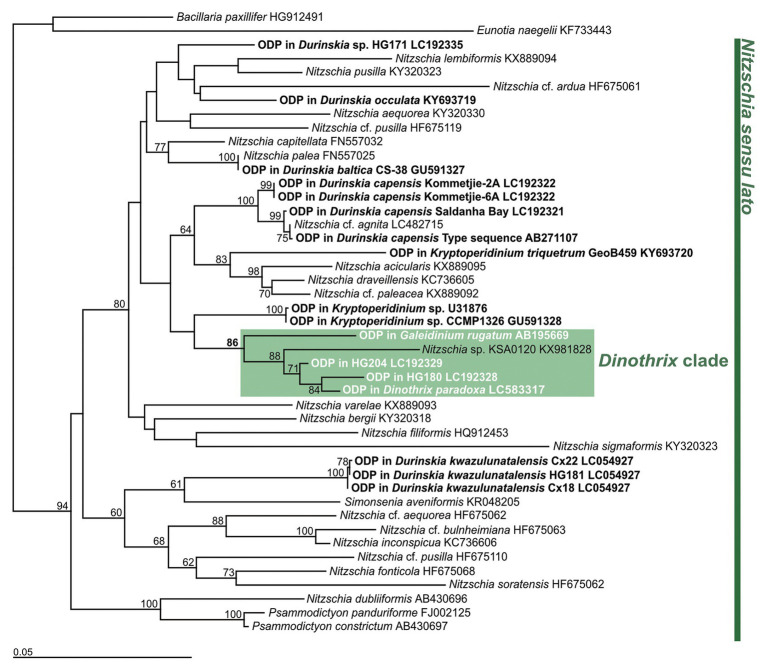
Two temporary-cultured dinotoms collected from natural samples. **(A)** A non-motile cell of *Gymnodinium quadrilobatum*; **(B)** A swimming cell of *D. paradoxa*. Arrowhead = Eyespot; **(C)** A non-motile cell of *D. paradoxa*; **(D)** Dividing cells of *D. paradoxa*. Two daughter cells produced in the previous cell division did not transformed into swimming cells, and started dividing newly; **(E-H)** The thecal plate tabulation of *D. paradoxa via* fluorescence microscopy (FM).

A single non-motile cell of *Gy. quadrilobatum* (Figure 3A) was isolated from the natural sandy samples (Table 1). The isolated species formed a four-leaf clover shaped non-motile cell (Figure 3A), which is a morphology specific to *Gy. quadrilobatum* (Horiguchi and Pienaar, 1994). We observed that this species, under our lab conditions, first divided to a maximum of up to 100 cells, although the frequency of cell divisions gradually decreased. At the same time, the daughter cells and the plastids started decreasing in size. All cells died 2.5 months after the isolation.

Around 100 cells of *D. paradoxa* (Figures 3B–D) were collected from a tidal pool when they were forming a bloom (Table 1). These cells were identified as *D. paradoxa* based on the observations of Horiguchi (1984). In this study, we collected the samples exactly from the same tidal pool where Horiguchi (1984) re-discovered this species. Collected samples were motile cells (Figure 3B), but during transportation to the laboratory, most of them settled on the bottom of the container, forming non-motile cells (Figure 3C). Daughter cells often maintain the non-motile phase after cell division and start dividing (Figure 3D). These cells were isolated and cultured in our lab, but gradually decreased in size, similarly to *Gy. quadrilobatum*. All the cells died several weeks later.

### Morphological Descriptions of *Dinothrix paradoxa* and Two Novel Dinotoms

#### Thecal Plates of *Dinothrix paradoxa*

Detailed cell morphology, cell ultrastructures, life cycle, and the pigment composition of *D. paradoxa* have been described by Pascher (1914, 1927) and Horiguchi (1984). Horiguchi (1984) identified that the swimming cells possessed thecal plates with the following plate tabulation: 4′, 2a, 7″, 5c, 4s, 5‴, and 2‴′ (Hoppenrath et al., 2014). No photographic data of the plate tabulation were shown in these studies; therefore, the thecal plate tabulation of swimming cells are shown in Figures 3E–H.

#### Cell Morphologies of Strains HG180 and HG204

**Figure 4:**
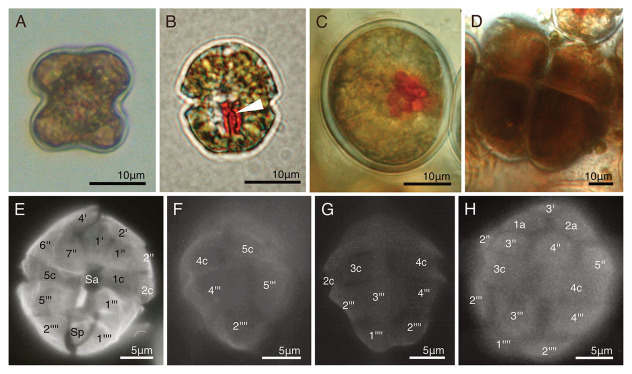
A culture-established novel dinotom, *Dinothrix phymatodea* sp. nov. (strain HG180). **(A)** A non-motile cell of *Dinothrix phymatodea* (strain HG180) under light microscopy (LM). Arrowhead = Eyespot; **(B)** A non-motile cell of *D. phymatodea* under scanning electron microscopy (SEM); **(C)** A swimming cell of *D. phymatodea* under LM. Arrowhead = Eyespot; **(D)** A swimming cell of *D. phymatodea* under SEM. No thecal plates were observed; **(E)** The cell wall of non-motile cells consisted of three materials; a dark-smooth wall (asterisk), a transparent-outer wall with nodes (two asterisks), and several thin membranes (arrowhead); **(F)** Plastids of *D. phymatodea* under FM; **(G,H)** Two nuclei of *D. phymatodea* under confocal laser scanning microscopy (CLSM) in the interphase **(G)** or before the cell division **(H)**. Diatom nucleus transformed to a string-like shape (arrows) in the interphase and to a round (DiaN) before the cell division. DinoN = Dinoflagellate nucleus; **(I)** A non-motile cell of *D. phymatodea* under TEM. A cell contained two nuclei derived from dinoflagellate (DinoN) or diatom (DiaN). The crystal-like materials **(C)** increased the amount, if the daughter cells did not transform to swimming cells. L, Lipid; P, Diatom plastid; and S, Starch. Asterisk = A nucleolus of dinoflagellate nucleus; **(J,K)** A symbiosome membrane (arrows) separated the diatom compartment from the host dinoflagellate cytoplasm. DiaM, diatom mitochondrion; DiaN, diatom nucleus; DinoM, dinoflagellate mitochondrion; ER (arrowhead), diatom ER; P, diatom plastid; and Py; pyrenoid. Asterisk = A nucleolus of diatom nucleus; **(L)** Diatom plastid membranes. ER, diatom ER, PM, periplastid membrane. Bar = 500 nm.

Both non-motile cells and swimming cells of strain HG180 were almost round or slightly ovoidal (Figures 4A–D). Non-motile cells (*n* = 10) measured 44.8 μm (±13.5 μm) in diameter, 19.9 μm in length (±1.5 μm), and 17.6 μm in width (±2.1 μm, *n* = 3) in the motile phase (*n* = 3). No cingulum, sulcus, and flagella were observed in the non-motile phase (Figures 4A,B). The cell wall in the non-motile phase was thick, had no thecal plate-like structures, and was composed of two types of inner and outer walls: a dark-colored smooth inner wall covered by a semi-transparent outer wall (Figures 4A,E). These walls were often covered with several mucus-like thin layers (Figure 4E). The semi-transparent outer wall had more than 100 small round nodes on the surface (Figures 4A,B). Thecal plates were not observed in the motile phase (Figure 4D). The 10–20 diatom plastids were brownish-yellow in color (Figures 4A,C), string-like to branched-shape, and distributed in the peripheral region of the cell (Figure 4F). An oblong small red eyespot was located in the middle of the cell in the non-motile phase (Figure 4A), while swimming cells had an obvious eyespot with a hook-like extension along the sulcus (Figure 4C). A single cell contained two eukaryotic nuclei: a host dinoflagellate nucleus with condensed chromosomes (dinokaryon) and a small diatom nucleus (Figures 4G–I). The diatom nucleus changed the morphology from a dispersed string-shape in the interphase (Figure 4G), to a round shape prior to cell division (Figure 4H), as reported in other dinotoms (Tippit and Pickett-Heaps, 1976; Yamada et al., 2019). The ultrastructure of non-motile cells was typical for dinotoms: in the cytoplasm of the dinoflagellate, a dinokaryon, mitochondria, starch granules, and lipids were observed (Figure 4I). Old cells of strain HG180 often produced crystal-like unknown materials (Figures 4A,I), which might be related to the dinoflagellate lysosome, the accumulation body (Zhou and Fritz, 1994). In the diatom compartment, a nucleus, mitochondria, ER, and plastids were observed (Figures 4J,K), which were all separated from the host cytoplasm by a symbiosome membrane (Figures 4J,K). Diatom plastids had a typical diatom shape, being composed of three thylakoids with an internal pyrenoid (Figure 4J) and surrounded by an ER and a periplastid membrane (PM), in addition to two typical plastid envelopes (Figure 4L).

**Figure 5:**
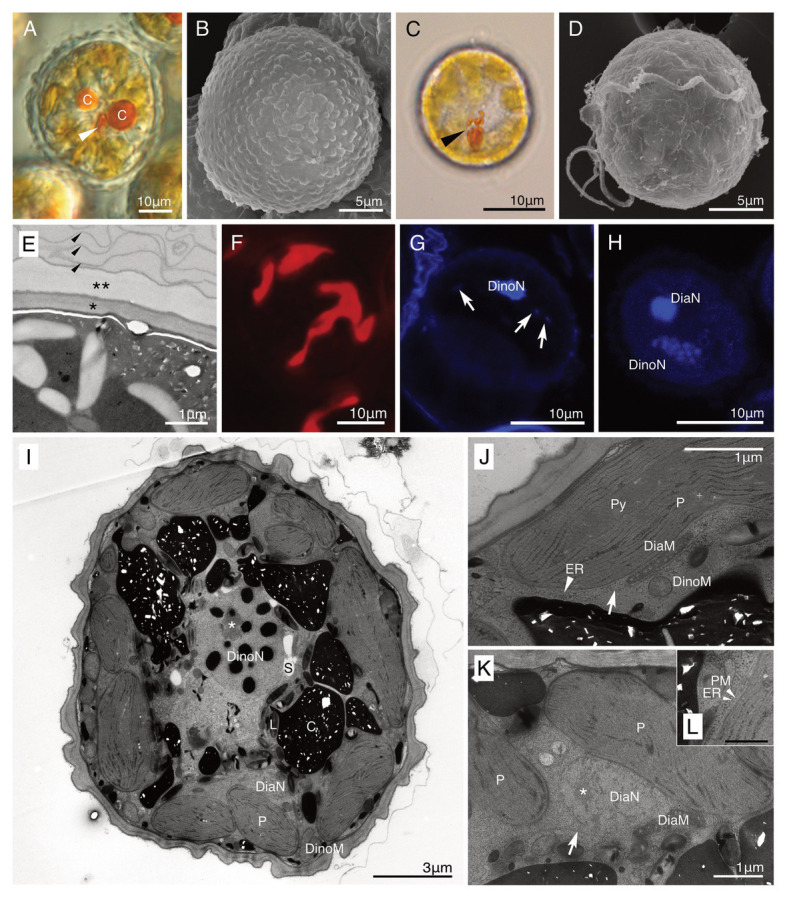
A culture-established novel dinotom, *Dinothrix pseudoparadoxa* sp. nov. (strain HG204). **(A)** A non-motile cell of *Dinothrix pseudoparadoxa* (strain HG204) under LM; **(B)** A non-motile cell of *D. pseudoparadoxa* under SEM; **(C)** A swimming cell of *D. pseudoparadoxa* under LM. Arrowhead = Eyespot; **(D)** A swimming cell of *D. pseudoparadoxa* under SEM. No thecal plates were observed; **(E)** The cell wall of non-motile cells consisted of two materials; a dark-winkle wall (asterisk), and several membranes (arrowhead); **(F)** Plastids of *D. pseudoparadoxa* under FM; **(G,H)** Two nuclei of *D. pseudoparadoxa* under CLSM in the interphase **(G)** or before the cell division **(H)**. Diatom nucleus transformed to a string-like shape (arrows) in the interphase and to a round (DiaN) before the cell division. DinoN, dinoflagellate nucleus; **(I)** A non-motile cell of *D. pseudoparadoxa* under TEM. A cell contains two nuclei derived from dinoflagellate (DinoN) or diatom (DiaN). L, Lipid; P, Diatom plastid; and S, Starch; **(J)** Crystal-like materials **(C)** were stocked in the dinoflagellate cytoplasm; **(K,L)** A symbiosome membrane (arrows) separated the diatom cytoplasm from the host dinoflagellate cytoplasm. ER, diatom ER, DiaM, diatom mitochondrion, DiaN, diatom nucleus, P, diatom plastid, PM, periplastid membrane, and Py, pyrenoid. Asterisk = A nucleolus of diatom nucleus.

**Figure 6:**
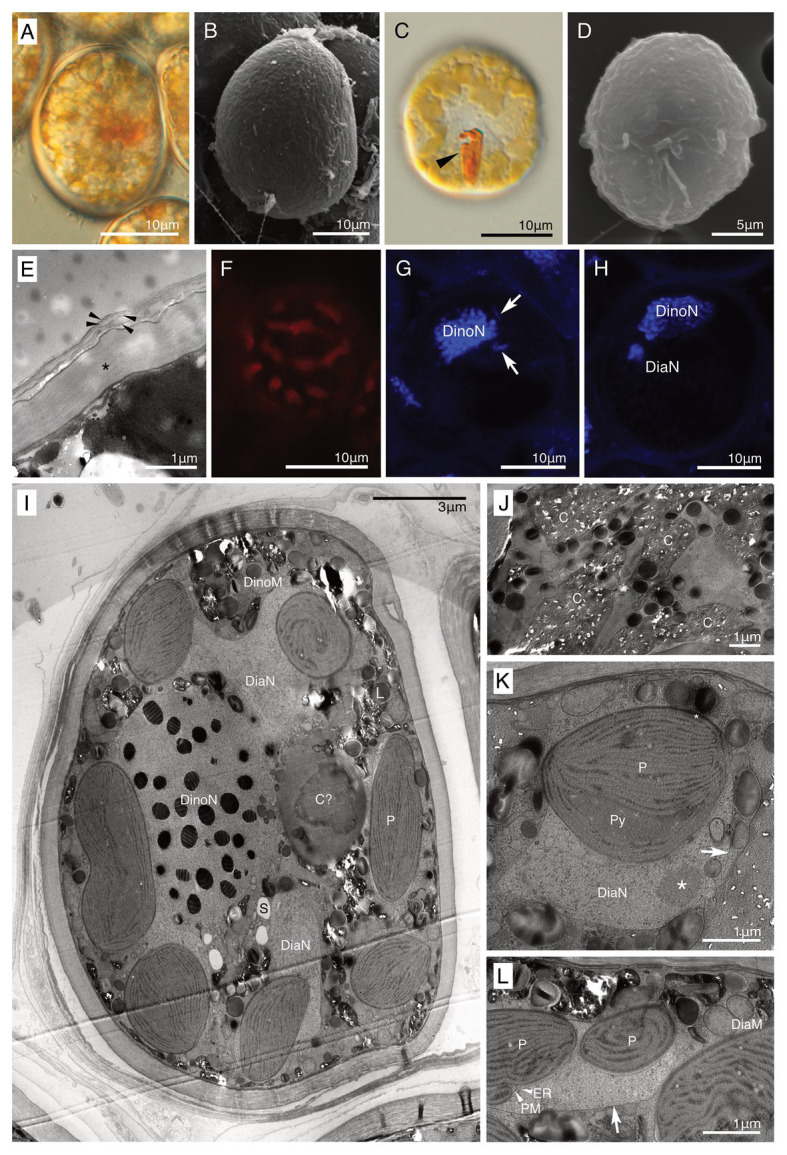
A membrane transport system between the host dinoflagellate and the ODP. **(A)** A diatom Golgi (DiaGolgi) was observed; **(B,C)** Small vesicles (arrows) were produced from the diatom ER. A protrusion (arrowhead) connected with the ER. Some of these vesicles were transported into the dinoflagellate cytoplasm side (asterisk). ER, diatom ER; P, diatom plastid; and PM, periplastid membrane. **(D)** Small vesicles (arrows) attached with the symbiosome membrane. A protrusion (arrowhead) connected with the symbiosome membrane.

Both non-motile and motile cells of strain HG204 were ovoidal (Figures 5A–D). The non-motile cells (*n* = 10) were 42.7 μm long (± 11.58 μm) and 29.6 μm wide (± 3.25 μm), while the swimming cells (*n* = 3) were 19.3 μm long (± 2.7 μm) and 15.8 μm wide (±3.3 μm). No cingulum, sulcus, and flagella were observed in the non-motile phase (Figures 5A,B). The semi-transparent thick cell wall was composed of multiple thin layers (Figures 5A,E) and was smooth under LM (Figure 5A), but subtle corrugations were observed under SEM (Figure 5B). Non-motile cells were often surrounded by several thin wall layers (Figure 5E). Thecal plates were neither observed in the non-motile (Figure 5B) nor the swimming cells (Figure 5D). The obvious eyespot could not be observed in the non-motile phase (Figure 5A), but in the motile phase, a hook-like, red eyespot appeared along the sulcus (Figure 5C). There were 10–20 diatom plastids, and they were all brownish-yellow in color (Figures 5A,C), string-shaped to oval, and distributed near the cell surface (Figure 5F). A single cell contained two eukaryotic nuclei: a typical dinokaryon with condensed chromosomes and a diatom nucleus (Figures 5G,H). The diatom nucleus changed the morphology from a dispersed string-shape in the interphase (Figure 5G) to a round shape prior to cell division (Figure 5H), similarly to other dinotoms (e.g., Figures 4G,H). The ultrastructure was typical for dinotoms: in the cytoplasm of the dinoflagellate, a dinokaryon, mitochondria, starch granules, and lipids were observed (Figure 5I). Old cells of HG204 also produced crystal-like materials (Figure 5J), similar to those of HG180. In the diatom compartment, a diatom nucleus, mitochondria, and ER were commonly observed (Figures 5K,L). A diatom Golgi was also found (Figure 6A), which has hardly been observed before, and only reported once in *Blixaea quinquecornus* (Horiguchi and Pienaar, 1991). Diatom plastids had a typical diatom shape, being composed of three thylakoids with an internal pyrenoid (Figure 5K), and surrounded by ER and a PM, in addition to typical two plastid envelopes (Figures 5L, 6B). We also observed that transport vesicles were produced by the diatom ER (Figures 6B,C), attached to the symbiosome membrane (Figure 6D), and transferred into the dinoflagellate cytoplasm (Figure 6C).

### Life Cycles of Strains HG180 and HG204

**Figure 7:**
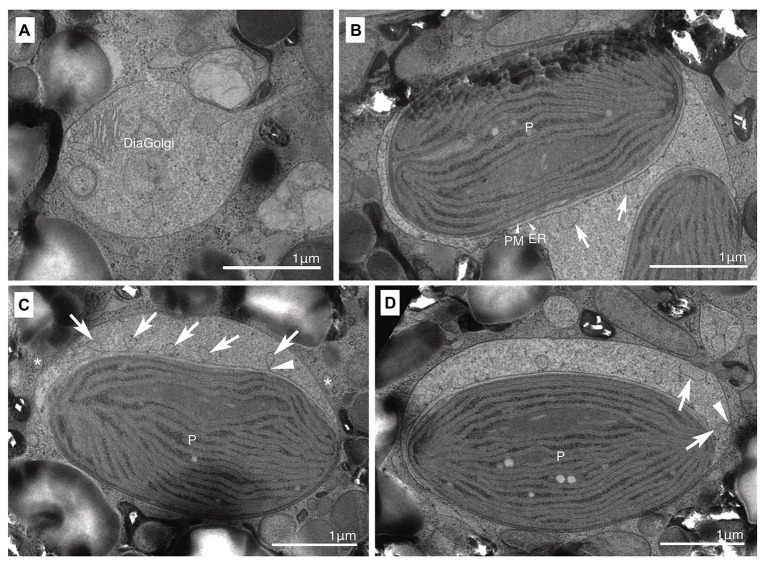
A comparison of the life cycles with two novel dinotoms, *Dinothrix phymatodea* sp. nov. (strain HG180) and *D. pseudoparadoxa* sp. nov. (strain HG204). **(A)** A dividing cell of *Dinothrix phymatodea* (strain HG180) under LM; **(B)** A dividing cell of *D. phymatodea* under SEM; **(C)** A dividing cell of *D. pseudoparadoxa* (strain HG204) under LM; **(D)** A dividing cell of *D. pseudoparadoxa* under SEM; **(E)** *D. phymatodea* consisting a cell clump; and **(F)** *D. pseudoparadoxa* consisting a cell clump.

The non-motile cell phases of HG180 and HG204 were their dominant phase throughout the life cycle. Cell division of both species occurred during the non-motile phases (Figures 7A–D), and the swimming cells never divided. The dividing rates per day were determined as 0.79 (HG180, *n* = 6) and 0.69 (HG204, *n* = 11). They usually remained in the non-motile phase after cell division, and formed cell clumps, consisting of two to several dozen cells, which could float and drift (Figures 7E,F). Swimming cells hardly appeared: swimming cells were only observed when these species were inoculated into fresh medium, or were cultured under stress conditions (e.g., high light or low temperature), but they could only persist for several hours, and settled on the bottom of the vessel within a day and transformed to non-motile cells.

## Discussion

### Five Non-motile Dinotoms Are Close Relatives Within Dinotoms

The molecular phylogeny of host dinoflagellates shows that these five non-motile dinotoms represent a clade with 100% bootstrap support, within the family Kryptoperidiniaceae (Figure 1; Supplementary Figure 1). This is supported by their common life cycles (Table 2). The current taxonomic categorization of the genus for these five non-motile dinotoms should be debated, because three of them are independently classified into different genera (Pascher, 1914; Horiguchi and Pienaar, 1994; Tamura et al., 2005). Using LM observation, *D. paradoxa* was described as a new species of a new genus in 1914, based on the cell morphology and characteristic life cycle (Pascher, 1914). Horiguchi (1984) re-discovered this species, and identified it as possessing ODPs, based on the ultrastructure and the pigment composition. However, its phylogenetic position has not been confirmed until this study. Ten years after the re-discovery of *D. paradoxa*, *Gy. quadrilobatum* was described as a novel species with ODPs, but unfortunately lacking genetic information (Horiguchi and Pienaar, 1994). This species was assigned into the genus *Gymnodinium*, of the family *Gymnodiniaceae*, based on morphology of the motile stage (Horiguchi and Pienaar, 1994). However, it is now clear from our molecular phylogenies that this species does not belong to the genus *Gymnodinium*. Another benthic dinotom, *Ga. rugatum*, was discovered in 2005, and described as a new species in a new genus, of the family Kryptoperidiniaceae, using sequences of host 18S rDNA and the diatom *rbc*L gene (Tamura et al., 2005). Considering the above taxonomic history of non-motile dinotoms, there are two possible taxonomic options for their genetic nomenclature: the first is to create three new genera for *Gy. quadrilobatum*, HG180 and HG204 separately, based on their diverse cell morphologies. Another is to integrate all the species belonging to the non-motile dinotom clade into one genus. We think that the latter option is reasonable, because of the shared life cycle and their phylogenetically close relationships. We conclude that these five non-motile dinotoms should be combined to the oldest genus among them, *Dinothrix*. Here, we re-define the genus *Dinothrix* and transfer the previously-described species, *Ga. rugatum* and *Gy. quadrilobatum* to genus *Dinothrix*.

**Table 2:** The life cycle of *Dinothrix* spp. and the sister group *Kryproperidinium* spp.

Species name	Domestic phase	Division phase	Appearance timing of swimming cell	Plate tabulation of swimming cell	Non-motile cellular form
*Dinothrix paradoxa*^1^^,^^2^^,^^3^^,^^4^	Non-motile	Non-motile	As the surrounding environment changes	4', 2a, 7'', 5c, 4 s, 5''', 2''''	Filamentous but limited to 2–10 cells
*Dinothrix phymatodea*^5^	Non-motile	Non-motile	As the surrounding environment changes	Athecate	Two to several dozen of round cell clump
*Dinothrix pseudoparadoxa*^5^	Non-motile	Non-motile	As the surrounding environment changes	Athecate	Two to several dozen of round cell clump
*Dinothrix quadrilobata*^6^	Non-motile	Non-motile	In each division	Athecate	Single cell form
*Dinothrix rugata*^7^	Non-motile	Non-motile	In each division	Athecate	Single cell form
*Kryptoperidinium* spp.^8^^,^^9^	Swimming	Swimming/non-motile	In each division in motile phase^10^	4'(3'), 2a, 7'', 5c (4c), 4 s, 5''', 2''''	Up to six cells

1 Pascher (1914);

2 Pascher (1927);

3 Horiguchi (1984);

4 Hoppenrath et al. (2014);

5 This study;

6 Horiguchi and Pienaar (1994);

7 Tamura et al. (2005);

8 Figueroa et al. (2009);

9 Gottschling et al. (2019).

10 It is unknown whether divided non-motile cells transform to swimming cells.

#### *Dinothrix Pascher* emend. N. Yamada and T. Horiguchi

##### Description

The original description is given in Pascher (1914), benthic dinoflagellates in the family Kryptoperidiniaceae of the order Peridiniales, species exhibit non-motile cells without flagella as the dominant phase, swimming cells appear after cell division, and plastids are derived from diatoms.

##### Type Species

*Dinothrix paradoxa* Pascher (GenBank accession numbers are LC583317 and LC583318).

#### *Dinothrix quadrilobata* (T. Horiguchi and R. N. Pienaar) R. Onuma and T. Horiguchi comb. nov.

##### Basionym

*Gymnodinium quadrilobatum* T. Horiguchi and R. N. Pienaar in Horiguchi and Pienaar (1994, P238, Figures 1–22).

##### Type Locality

Amanzimtoti, Kwazulu-Natal Province, South Africa. Nuclear-encoded 18S rDNA sequence of the sample collected from Japan is available (GenBank accession number is LC583319).

#### *Dinothrix rugata* (M. Tamura and T. Horiguchi) N. Yamada and T. Horiguchi comb. nov.

##### Basionym

*Galeidinium rugatum* M. Tamura and T. Horiguchi in (Tamura et al., 2005, P661, Figures 1–4).

##### Type Locality

Mecherchar Island, the Republic of Palau. Sequences of nuclear-encoded 18S rDNA and plastid-encoded *rbc*L gene are available (GenBank accession numbers AB195668 and AB195669).

Interestingly, *Dinothrix* spp. exhibit slightly different life cycles, allowing us to categorize two types by the timings of the appearance of swimming cells (Table 2). The first type is observed in *D. quadrilobata* and *D. rugata*. Without exception, their daughter cells transform into two swimming cells after cell division. Therefore, their non-motile cells invariably exist as a sessile single cell form on the bottom culture vessels. Another type is represented by *D. paradoxa*, and the two novel *Dinothrix* spp. strains HG180 and HG204: their swimming cells appear only if the environment changes or under inappropriate culture conditions, such as low temperature or high light. Otherwise, they remain in the non-motile phase after cell division. As a consequence, these dinoflagellates divide continuously in the non-motile phase, forming cell clumps that often float and drift in the culture medium. Considering that at least *D. paradoxa* and HG204 were originally collected as they were in the swimming form, it is possible that the rare appearance of their swimming cells is unnatural, and might require triggers, e.g., the rise and fall of the tides, to produce swimming cells after every cell division.

Non-motile cells are also observed in the genus *Kryptoperidinium*, the sister genus of *Dinothrix* spp., and the species commonly collected from marine or brackish coastal regions. For example, the dominant life phase of *Kryptoperidinium cf. foliaceum* (strains Baiona A3, B1, or B9; Figueroa et al., 2009) is the motile phase. However, this species is able to produce non-motile cells during the vegetative cycle. These non-motile vegetative cells exist in abundance in cultures, and perform the cell division, even though the swimming cells also undergo cell division. In some cases, a cell clump consisting of up to six cells are produced from a single non-motile cell (Figueroa et al., 2009). This observation indicates that the primitive life cycle of *Dinothrix* spp. was originally shared with *Kryptoperidinium* spp., and it developed in *Dinothrix* species in order to adapt the benthic environments after *Kryptoperidinium* spp. and *Dinothrix* spp. diverged.

### Descriptions of Two Novel *Dinothrix* Species

Even though the life cycle and molecular phylogeny indicate that the non-motile dinotoms belong to the same genus, it is possible to distinguish them morphologically at the species level: ovoidal with smooth cell walls (*D. paradoxa* and HG204; Pascher, 1914, 1927; this study), clover-shaped cell with smooth walls (*D. quadrilobata*; Horiguchi and Pienaar, 1994), pyramid-shaped cell with wrinkled walls (*D. rugata*; Tamura et al., 2005), and round with a number of surface nodes (HG180; this study). *Dinothrix paradoxa* and HG204 can be distinguished by the following characteristics: the former species forms filamentous cell clumps, consisting of limited numbers of between 2 and 10 cells (Pascher, 1927; Horiguchi, 1984), while the cell clumps of the latter species consist of two to several dozens of cells. The swimming cell stages are also distinguishable by the presence (in *D. paradoxa*) or absence (in HG204) of thecal plates. Another distinction between these two species is a difference in cultivability (HG204 is cultivable, *D. paradoxa* is uncultivable), which might be caused by differences in their ODP evolutionary stages (discussed in the next section). Considering the characteristic morphologies and the phylogenetic positions, we describe HG180 and HG204 as independent novel species of genus *Dinothrix*: *Dinothrix phymatodea* sp. nov. and *D. pseudoparadoxa* sp. nov., respectively.

It is important to note that we only observed thecal plates in *D. paradoxa* (Figures 3E–H). In our SEM, TEM, and fluorescence microscopy (FM) observations, any thecal plates were not found in the other four *Dinothrix* species as in previous studies (Horiguchi and Pienaar, 1994; Tamura et al., 2005). Since all other dinotoms with the exception of these four *Dinothrix* species possess thecal plates, there is a possibility that all *Dinothrix* species have, in fact, very thin thecal plates, which were unable to be observed *via* microscopic preparations used in this and previous studies. But in this study, we treat *Dinothrix* spp. except for *D. paradoxa* as athecate dinoflagellates based on our and previous observations.

#### *Dinothrix phymatodea* N. Yamada and T. Horiguchi sp. nov.

##### Description

Non-motile round cells, covered with thick semi-transparent, concavo-convex wall with a number of nodes; 44.8 μm in diameter in the non-motile phase, and 19.9 μm long and 17.6 μm wide in the swimming phase; typical dinokaryon spherical; a red eyespot with ovoidal shape in the non-motile phase, hook-like extension in the swimming phase; possession of diatom organelles derived from a Nitzschoid, including a nucleus, mitochondria, ER, and plastids; diatom plastids brownish-yellow, discoidal, 10–20 in number, located in the periphery of the cell; only non-motile cells can divide; the daughter cells can continuously divide without producing the motile cells and resultant cell clumps can be large; naked swimming cells; dinoflagellate marine, sand-dwelling; and sequences of dinoflagellate nuclear-encoded 18S rDNA (GenBank accession LC054946), diatom nuclear-encoded 18S rDNA (LC192339), and diatom plastid-encoded *rbc*L gene (LC192328) are available.

##### Holotype

The SEM stub of strain HG180 used to take photographs used in this study was deposited in the herbarium of the Faculty of Science, Hokkaido University, Japan (No. SAP 115587).

##### Type Locality

26°06′51.9″N, 127°44′35.8″E; sandy bottoms in the coral flat, Hanashiro, Okinawa Prefecture, Japan.

##### Etymology

*phymatodea* named after its knobbed cell wall in Latin.

#### *Dinothrix pseudoparadoxa* N.Yamada and T.Horiguchi sp. nov.

##### Description

Non-motile ovoidal cells, covered with thick semi-transparent, smooth wall with subtle wrinkle-patterns; 42.7 μm long and 29.6 μm wide in the non-motile phase, and 19.3 μm long and 15.8 μm wide in the swimming phase; typical dinokaryon spherical; a red eyespot with hook-like extension in the swimming phase; possession of diatom organelles derived from a Nitzschoid, including a nucleus, mitochondria, ER, and plastids; diatom plastids brownish-yellow, discoidal, 10–20 in number, located in the periphery of the cell; only non-motile cells can divide; the daughter cells can continuously divide without producing the motile cells and the resultant cell clumps can be large; naked swimming cells; dinoflagellate marine, tidal pool living; and sequences of dinoflagellate nuclear-encoded 18S rDNA (GenBank accession LC054947), diatom nuclear-encoded 18S rDNA (LC192340), and diatom plastid-encoded *rbc*L gene (LC192329) are available.

##### Holotype

The SEM stub of strain HG204 used to take photographs used in this study was deposited in the herbarium of the Faculty of Science, Hokkaido University (No. SAP115588).

##### Type Locality

30°56′30.1''S, 30°18′16.6″E; sandy tidal pools on a sandy beach, Marina Beach, KwaZulu-Natal Province, South Africa.

##### Etymology

*pseudo-paradoxa* named after its similar morphology to *D. paradoxa* in Latin.

### *Dinothrix* spp. Might Possess a Variety of Origins and Integration Stages of ODPs

It has been reported that dinotoms maintain diverse origins of ODPs (Horiguchi and Takano, 2006; Takano et al., 2008; Yamada et al., 2017). At present, at least 14 species of diatoms, belonging to six genera: *Nitzschia*, *Simonsenia*, *Chaetoceros*, *Discostella*, *Cyclostephanos*, and *Cyclotella*, were confirmed as tertiary plastids in dinotoms (Čalasan et al., 2017; Yamada et al., 2019). The diverse origins of ODPs indicate that timing of acquiring current ODPs varied depending on the host dinoflagellate. This resulted in evolutionary variation in the integration stages in the ODPs, from kleptoplasty to permanent endosymbiosis (Yamada et al., 2019). Yamada et al. (2017) have previously suggested that the ODPs among *Dinothrix rugata*, *D. phymatodea*, and *D. pseudoparadoxa* originated from a single *Nitzschia* species, based on the ODPs monophyly, inferred from the diatom 18S rDNA and *rbc*L gene. The monophyly of ODPs in *Dinothrix* spp. is again highly supported in this study, with 86 or 99% bootstrap values by PhyML or IQTREE. However, the sequences of their *rbc*L gene show significant diversity; differences of 1.179–4.348%, compared to the ODP of *D. paradoxa*. This genetic diversity indicates that *Dinothrix* spp. possess multiple species of *Nitzschia* diatoms, although there is no morphological evidence for this speculation, because these diatoms’ frustules have already been lost when they were ingested by host dinoflagellates. Our failure in establishing cultures of *D. paradoxa* and *D. quadrilobata* further implies that the integration stages of these *Nitzschia*-derived ODPs are different from other cultivable *Dinothrix* spp. *D. quadrilobata* increased its cell numbers, from a single cell to approximately 100 under lab conditions, before the daughter cell and the plastid sizes became smaller, causing eventual cell death. *Dinothrix paradoxa* could be maintained in the natural crude culture for a few weeks, but eventually the cells decreased in size and died off. Even though the same culture medium and conditions were used, we were able to establish stable *D. rugata*, *D. phymatodea*, and *D. pseudoparadoxa* strains (also found in Tamura et al., 2005). We confirmed that *D. rugata*, *D. phymatodea*, and *D. pseudoparadoxa* possess permanent ODPs, which could synchronously divide when the host cell divides (Tippit and Pickett-Heaps, 1976; Figueroa et al., 2009). Hypothetically, *D. paradoxa* and *D. quadrilobata* could possess temporary ODPs, which cannot be permanently maintained by the host dinoflagellates, due to the lack of synchronous division (Yamada et al. 2019). However, to validate this, feeding experiments and long-term observations of *D. paradoxa* and *D. quadrilobata* are required.

It is notable that *Dinothrix* spp. possess ODPs, which are all closely-related to an undescribed free-living *Nitzschia* species (strain KSA120). Considering the wide geographic range of *Dinothrix* spp. (Table 1), it is very surprising that *Dinothrix* spp. show a strict prey preference for diatoms. If *D. paradoxa* and *D. quadrilobata* do possess temporary ODPs, they would have to constantly feed on the free-living diatom, which can be critical if the right diatoms are not available within their habitats. The reasons for *Dinothrix* spp. acquiring on a specific clade of diatoms is not yet known, but we can propose three possibilities: the close relatives of strain KSA120 are abundant and are common diatoms found in benthic environments worldwide; the significant amount of gene transfer from the clade of *Nitzschia* spp. to the host nucleus had already occurred; or specific metabolic connections between the hosts and their ODPs were established among the clade of *Nitzschia* and *Dinothrix* spp., and other diatoms cannot be used for photosynthesis in *Dinothrix* spp. Particularly for the second possibility, some transcriptomic analyses which have targeted kleptoplastic dinoflagellates or dinotoms revealed that very few or no genes have transferred to the host dinoflagellates from their current favorite prey microalgae (Wisecaver and Hackett, 2010; Hehenberger et al., 2016; Hongo et al., 2019). In light of these previous studies, gene transfer might not have a significant effect on diatom selection in dinotoms. For the other two possibilities, it is essential to investigate the species compositions of their ecological communities or undertake physiological experiments in laboratory settings.

### A Membrane Transport System From ODPs to Host Dinoflagellates or *vice versa*

The cryofixed TEM samples of *D. phymatodea* and *D. pseudoparadoxa* show that there might be a membrane transport system of metabolites from the ODPs to the host cytoplasm or *vice versa*, although TEM observations cannot provide information on the metabolic pathways involved. The transport vesicles appear to be produced by the diatom ER and transferred to the dinoflagellate cytoplasm. It is also possible that these vesicles are produced by the host dinoflagellate and are transported into the diatom plastids through the symbiosome membrane. At present, it is impossible to determine the direction of vesicles. The symbiosome membrane is a membrane that separates the endosymbionts or organelle-retaining plastids, from the host cytoplasm. Functionally-similar membranes, which might be involved in the transport of ATPs, nutrients, and photosynthates, as well as in protecting the ingested heterogeneous organelles, symbionts, or parasites from the host lysosomes, are commonly observed, for example between *Rhizobium* bacteria and host leguminous plants (Remigi et al., 2016), between endosymbiotic *Chlorella* and host ciliate *Paramecium bursaria* (Kodama and Fujishima, 2005, the membrane is called as perialgal vacuole membrane), between parasite Apicomplexa and host mammal cells (Schwab et al., 1994, the membrane is called as parasitophorous vacuole membrane), and between the endosymbiotic dinoflagellate *Symbiodinium* and host corals or sea anemones (Peng et al., 2010). In the last case, proteomic analysis revealed that distinct transporters, receptors, ATP synthases, and cytoskeleton components of the host cells are located on the surface of the symbiosome membrane (Peng et al., 2010). The transcriptomic analysis of two dinotoms suggests that the 3-phospho glycerate, a carbon product of photosynthesis in plastids, and biotin (vitamin B_7_) can be transported *via* transporters located in the symbiosome membrane. This contributes to the hosts’ starch-synthesis pathway, or biotin is used directly by the host (Hehenberger et al., 2016). So far, other interacting pathways of ODPs and host cells are not known in dinotoms.

## Data Availability Statement

The datasets generated for this study can be found in the NCBI accession LC583317-LC583319.

## Author Contributions

NY and TH designed this project. TH isolated and established new strains of *Dinothrix phymatodea* and *D. pseudoparadoxa*. HS sampled, sequenced, and took the photos of *D. paradoxa*, and RO sampled, sequenced, and took the photo of *D. quadrilobata*. All other experiments including microscopic observations and constructing molecular phylogenies were performed by NY in TH or PK laboratories. NY wrote this manuscript, and all co-authors joined in the drafting processes. All authors contributed to the article and approved the submitted version.

### Conflict of Interest

The authors declare that the research was conducted in the absence of any commercial or financial relationships that could be construed as a potential conflict of interest.
