# Supplementary material for: Five Non-motile Dinotom Dinoflagellates of the Genus Dinothrix
Source: Front Plant Sci. 2020 Nov 19;11:591050. doi: 10.3389/fpls.2020.591050 (PMC7710806; doi:10.3389/fpls.2020.591050)
Supplement: Supplementary file 1 [file Image_1.pdf]

Supplementary Material

1 Supplementary Figures and Tables

1.1 Supplementary Figures

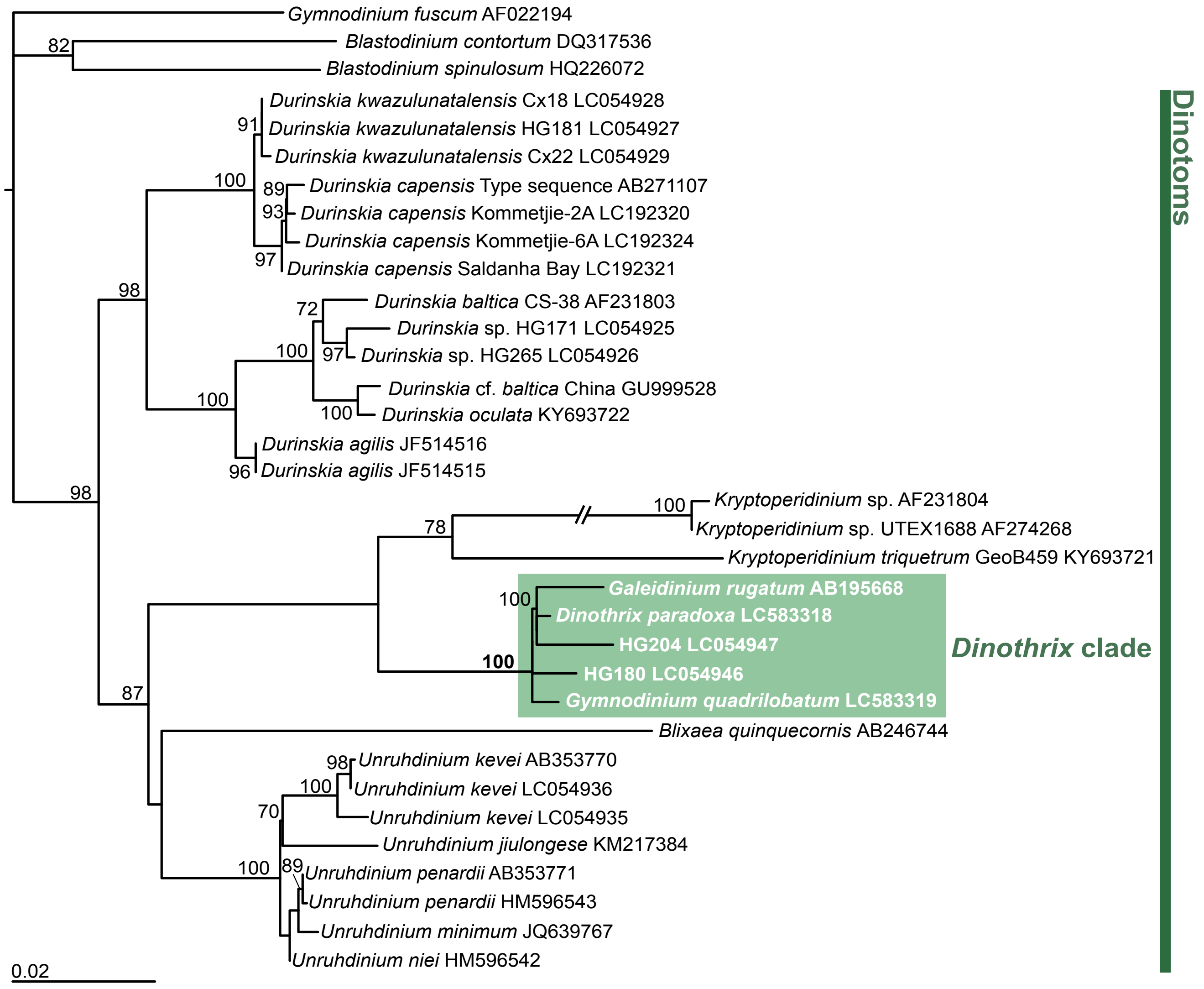

Supplementary Figure 1. Dinoflagellate tree constructed by IQTREE based on 18 rDNA. *Gymnodinium fuscum* (AF022194), *Blastodinium spinulosm* (HQ226072) and *Blastodinium contortum* (DQ317536) were used as outgroups. Numbers at the major nodes represent maximum

likelihood (1000 pseudoreplicates) bootstrap values. Only bootstrap values>70% are shown. GenBank accession numbers follow taxon names.

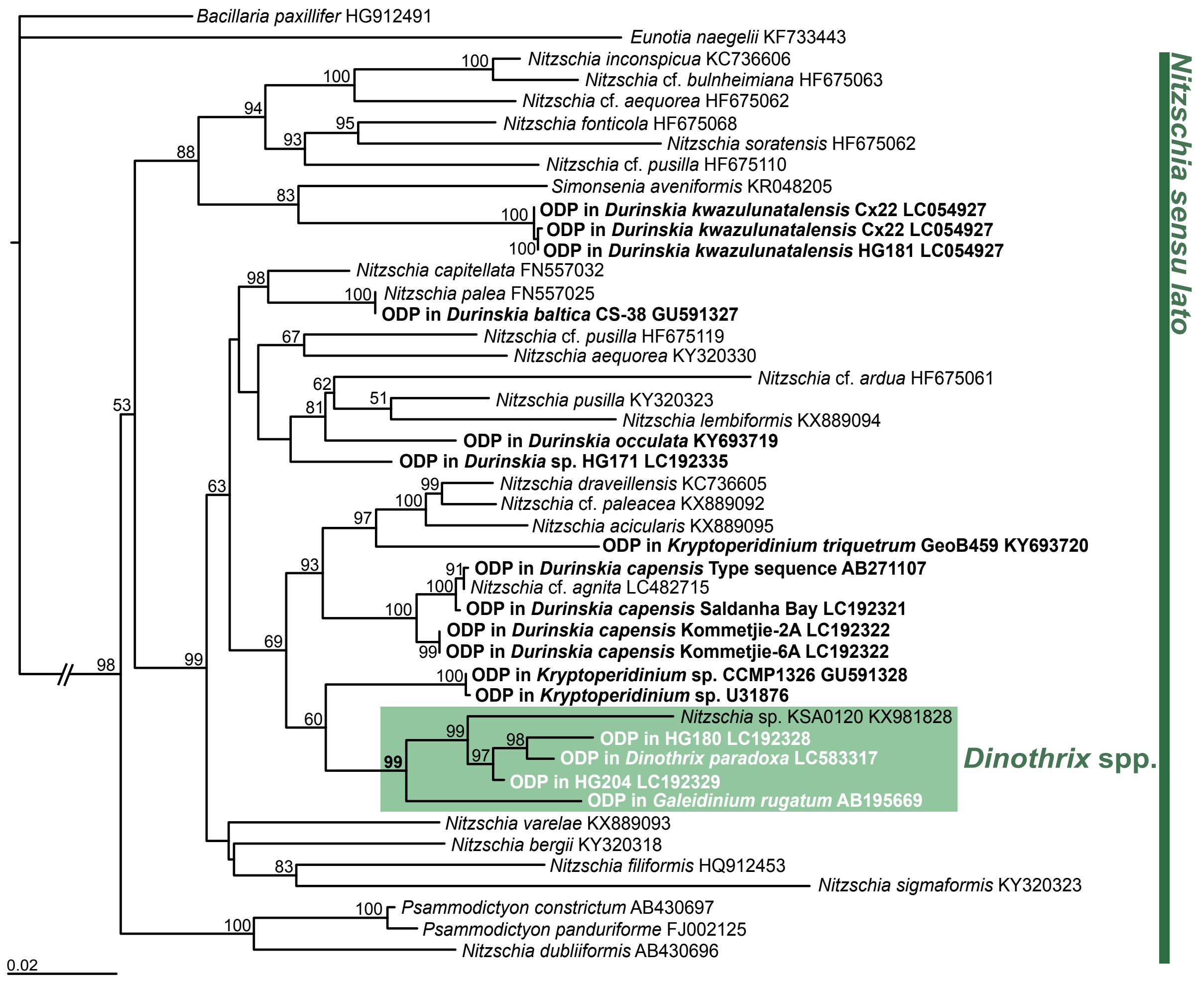

Supplementary Figure 2. Diatom tree constructed by IQTREE based on *rbc*L gene. *Bacillaria paxillifer* (HG912491) and *Eunotia naegelii* (KF733443) were used as outgroups. Bold type indicates the ODPs of dinotoms. Numbers on the major nodes represent maximum likelihood (1000 pseudoreplicates) bootstrap values. Only bootstrap values>50% are shown. GenBank accession numbers follow taxon names.

1.2 Supplementary Table.

Supplementary Table 1. A list of primer names and the sequences used for 18S rDNA of host dinoflagellates and the *rbc*L gene from their ODPs.

Primer name	Sequence 5' to 3'

SR1b	GATCCTGCCAGTAGTCATATGCTT
SR3	AGGCTCCCTGTCCGGAATC
SR2spin	CACTCAAGTTTCTGACCTATC
SR7	TCCTTGGGCAAATGCTTTCGC
SR4	AGGGCAAGTCTGGTGCCAG
SR9p	AACTAAGAACRGCCATGCAC
SR6	GTCAGAGGTGAAATTCTTGG
SR11	CGCTTACTAGGAATTCCTCG
SR8	GGATTGACAGATTGAGAGCT
SR12b	CGGAAACCTTGTTACGACTTCTCC
DiatrbcL1	TATATATTGCCTTTTTATTC
DiatrbcL3	AAACCACCTTTTAAACCTTC
DiatrbcL2	ACAGTAAAACCWAAATTAGG
DiatrbcL5	ATTTGACCACAGTGGATACC
DiatrbcL4	TGTAAATGGATGCGTATGT
DiatrbcL6	GTCTCACTATTCAAATACTC
